# Food provision to support improved nutrition and well-being of people experiencing disadvantage – perspectives of service providers

**DOI:** 10.1017/S1368980024000132

**Published:** 2024-01-15

**Authors:** Verena T Vaiciurgis, AK Clancy, KE Charlton, A Stefoska-Needham, EJ Beck

**Affiliations:** 1 School of Medical, Indigenous and Health Sciences, Faculty of Science, Medicine and Health, Building 41 Room 226, University of Wollongong, Wollongong, NSW 2522, Australia; 2 School of Health Sciences, University of New South Wales, Kensington, Australia

**Keywords:** Food, Nutrition, Socio-economic disadvantage, Diet-related health inequalities

## Abstract

**Objective:**

Diet quality is significantly impacted by social and environmental factors. People experiencing socio-economic disadvantage face inequitable barriers to accessing nutritious foods and health services, resulting in significant health disparities. This study aimed to explore the barriers faced by organisations that provide food support to people experiencing disadvantage as well as to identify potential strategies to enhance this support for improved well-being of clients.

**Design:**

Semi-structured interviews using an exploratory approach and inductive thematic analysis.

**Setting:**

Australia.

**Participants:**

Individuals from organisations involved in the provision of food support for people experiencing disadvantage aged ≥16 years.

**Results:**

Two major themes were identified from thirteen interviews. ‘Dignity and respect for clients’ serves as a guiding principle for food-related services across all organisations, while ‘food’ was a point of connection and a potential gateway to additional support pathways. Five additional subthemes included ‘food as a platform to reduce social isolation, foster connection and promote participation’, challenges with ‘servicing clients with diverse experiences and needs’, ‘dependence on staff and volunteers with varying knowledge and skillsets’, ensuring ‘adequate access to services, resources and facilities’ and ‘necessity of community collaboration’.

**Conclusions:**

This study highlights the unique position of organisations involved in food support to identify client-specific needs and implement broader holistic health support. Future interventions should prioritise dignity, respect and social connection in design. Organisations require an adequately trained, sustainable workforce, with shared or enhanced services, resources and facilities, and greater community coordination with other services to maximise effectiveness.

Socio-economic disadvantage refers to the limited access individuals or groups face to resources, opportunities, and social privileges due to their economic and social circumstances. Within Australia, those particularly vulnerable to experiencing deep and persistent socio-economic disadvantage include single person households and their children, individuals with lower levels of educational attainment, people living with long-term health conditions, disabilities or mental illnesses, people living in rural and remote communities and Aboriginal and Torres Strait Islander people^(1,2)^. This disadvantage is driven by a complex interplay of various and multidimensional factors, including intergenerational, individual and environmental influences and adverse life events such as job loss, housing stress, low level of educational attainment, low income, divorce/separation, long-term health issues, trauma, family and domestic violence and criminal convictions and/or prison incarcerations^(1,3)^.

Food security is a significant issue affecting the nutrition and health status of individuals who are unable to afford and/or access safe and nutritionally adequate food^(4)^. Food security encompasses six dimensions: food availability, physical and economic resources to access food, knowledge and resources for the safe utilisation of food (e.g. clean water/ storage, and preparation facilities) and stability or ongoing access to food availability, access and utilisation at all times, agency that refers to the capacity of individuals and groups to exercise voice and make decisions about their food systems and sustainability that refers to the long-term viability of the ecological and social bases of food systems^(5)^.

Socio-economic disadvantage is a key driver of food insecurity and health inequities, both in Australia and globally^(4,6)^. In Australia, charitable food systems, including food banks and not for profit food services, are the primary response to food insecurity^(6–8)^. While these services are being utilised by those facing the greatest need and offer valuable emergency food relief, it is now widely recognised they cannot meet increasing demand and address the root causes of chronic or long-term food insecurity^(6,9)^.

Suboptimal diets, including nutrient deficiencies and poor long-term dietary patterns, are aetiologically linked to chronic health conditions such as CVD, diabetes and cancer and are a major cause of mortality and disability globally, despite being a key modifiable risk factor^(10,11)^. This equates to one in five deaths associated with poor diet globally^(12)^.

Diet and nutrition are greatly affected by the social determinants of health with higher income, education and social status proportionately linked to improved health^(13)^. People experiencing socio-economic disadvantage face barriers to accessing adequate nutrition^(13)^, including unequal exposure to psychosocial stress and cultural, economic and environmental risk factors^(14–16)^ (e.g. physical environment, trauma exposure, appropriate storage and preparation facilities, income, food prices, self-efficacy, skills and knowledge).

People experiencing socio-economic disadvantage also experience disparities in accessing available, affordable and appropriate health services, exacerbating the diet-related health inequities^(17,18)^. Limited access to primary health services is associated with several negative consequences such as poor control of manageable chronic conditions (including later stage diagnosis)^(19,20)^ and missed opportunities for disease prevention that lead to significant costs for healthcare systems, estimated to be far greater than those incurred for the provision of preventative health services^(21)^. The Australian Government’s National Preventative Health Strategy 2021–2030 recognises the importance of holistic health and well-being, which encompasses individuals’ and communities’ physical, mental, social and cultural aspects^(22)^. The strategy emphasises the importance of establishing sustainable prevention systems to support overall health and reduce disease burden. Implementing effective preventative health measures holds substantial potential for economic benefits, estimated to increase Australia’s gross domestic product by $4 billion annually^(23)^. This is supported by systematic economic evaluations on the cost-effectiveness of public health^(24)^ and dietary interventions^(25,26)^ (e.g. nutrition policies and mandatory regulations) leading to sustained and larger-scale beneficial outcomes. While most organisations may be unable to address the root causes of chronic or long-term food insecurity, service providers, particularly charitable or low-cost government organisations, may be well placed to offer and/or initiate nutrition-related support (e.g. education). Such services have regular interactions with clientele, creating opportunities for care or referrals to experts, including in relation to nutrition. However, limited research is available on long-term strategies to effectively address modifiable dietary-related drivers of costly health inequities in disadvantaged populations^(27)^. This knowledge gap highlights a need for further investigation and innovative approaches to address the issue. The primary objective of this study was to explore the barriers faced by individuals providing food support to people experiencing disadvantage. Additionally, the study aimed to identify potential strategies that could enhance the support of nutrition-related health and well-being for people experiencing disadvantage.

Nutrition-related health refers to the potential for high-quality dietary intake and access to healthcare to minimise risks for non-communicable disease. This study is part of a larger research initiative, underpinned by participatory action research methodology, aiming to co-design targeted solutions to reduce health inequities related to diet. Other aspects of the research have engaged the primary stakeholders, namely people experiencing disadvantage (Food Science and Nutrition. In review).

## Methods

In-depth semi-structured individual interviews explored the perspectives of staff or volunteers from Australian organisations assisting in food support for people experiencing disadvantage aged 16 years and over. Participants who had previously completed an online survey with similar aims were invited to participate in an interview. To be eligible, the organisations needed to provide food and nutrition-related support (e.g. emergency food assistance; food relief and/or emergency and temporary accommodation, hostels for the homeless and transitional housing where food is provided). Given the wide scope of residential aged care and disability settings within Australia, these services were excluded. The study was fully approved by the University of Wollongong Human Research Ethics Committee (Approval number 2022/106), and written consent was obtained from both organisations and participants. The reporting of findings adhered to the guidelines outlined in The Consolidated Criteria for Reporting Qualitative Research^(28)^.

### Interviews

All sixty-eight participants who completed an online survey were invited to participate in a semi-structured individual interview conducted via videoconferencing platform (Zoom Video Communications Inc., 2023). The purpose of the interviews was an in-depth exploration of the data obtained from the survey. Interviews were conducted between July and September 2022, using an exploratory approach with predominately open-ended questions and probing (Table 1). This flexible approach allowed exploration of the lived experiences of the organisational staff and volunteers to identify their perceptions of factors influencing the dietary intake of clients, as well as identify potential strategies to better support their clients. Interviews were moderated by a single researcher with dietetics qualifications, with guidance from experienced qualitative researchers who also hold dietetics qualifications, including observation of interviews. Questions to staff members/volunteers were informed by a previous scoping review^(27)^ and included an inquiry about the nature of their organisation, the types of nutrition services provided, linked services and referral pathways, any nutrition-related concerns or requests of clientele, provision of nutrition education and identification of barriers and opportunities for organisations to improve/extend current support services (Table 1).

**Table 1:** Semi-structured interview question and probe guide

1.	How often do you/did you work at [your organisation]?
2.	What type of services does your organisation provide?
3.	Can you describe the food or nutrition services your organisation provides? Prompts what does that look like e.g. meals delivered, meals given in dining room, residential cooking/life skills programs – i.e. context sort of services/items do you provide and how do you do that, who does that?
4.	Have your clients ever requested an extension of these services or if you offer no services, have they requested services or information regarding nutrition? (requested nutrition-related services? - How are these requests handled? Referred elsewhere? Other options?
5.	Are you aware of any nutrition-related problems that clients may be experiencing (irrespective of if they request or not)? (prompts – obesity, high cholesterol, poor dentition, problems with purchase of food, cooking skills)
6.	Have you ever received any nutrition-related education/training or does your organisation provide any nutrition-related education/training for staff and volunteers?
7.	Are there any barriers for your organisation to provide the food or nutrition services mentioned above or extending these services? (Prompts infrastructure/equipment/facilities/budget/staffing/skills?)
8.	(Thinking of a perfect situation and thinking of the barriers of the organisation and the problems of the clients) If you had the opportunity to offer more services to help people have more healthful diets, what would you do differently? Prompt food services, meals, programs? 8B Whose responsibility do you think it is to ensure food and nutrition security in vulnerable populations? (health services, volunteer organisations, etc)
9.	What resources/support would you need to implement these services? Staff? Staff training? Cooking facilities?

## Data analysis

All interviews were conducted online, audio-recorded and transcribed verbatim by Zoom Video Communications Software and/or Otter.ai transcription software. All transcripts were checked for data authenticity, accuracy and consistency. All transcribed data were de-identified and thematically analysed using an inductive approach of the six-phase method described by Braun and Clarke^(29)^, utilising NVivo 12 qualitative software program. Each transcriptions was read independently by at least two researchers for familiarity (VV, AC and EB) to generate initial codes systematically across the datasets. These codes were then collated into candidate themes, which were reviewed using the two-level method to ensure accuracy and cohesion, then refined to generate a thematic map^(29)^. Identified themes were discussed with all members of the research team to reach a consensus of key themes. VV, AC and EB are all dietitians. VV is a doctoral student, and AC and EB are experienced qualitative researchers with doctoral qualifications. VV and AC have undertaken clinical work providing specific services to people experiencing disadvantage.

## Results

Thirteen interviews (twelve client facing) were undertaken with participants involved in food provision for people experiencing disadvantage within Australia (New South Wales 5, Victoria 5, South Australia 1, Queensland 1 and Western Australia 1, where New South Wales and Victoria are the most populous states). The thirteen participants were current employees within their organisations (male *n* 4, female *n* 9), all of whom were registered with the Australian Charities and Not-for-Profit Commission. This included religious organisations (*n* 8), locally run, neighbourhood centres and community services (*n* 4) and a food relief organisation servicing multiple charity groups (*n* 1). Prior to their current roles, participants reported diverse backgrounds, educational qualifications and experience which included chartered accountants, lived experience with disadvantage and accessing emergency food relief and/or food-related support (including previous volunteers and/or service users within their current organisation), hospitality workers, caseworkers and executive management. All reported receiving training in food safety only (*n* 13), with no formal education in nutrition.

Overall, participant interviews revealed two major themes. (Table 2) with five additional subthemes (Table 3) relevant to food and nutrition. The first major, overarching theme emphasised the importance of ‘dignity and respect for clients’ guiding all support in this context. Additionally, ‘food’ was recognised as more than just a fundamental human right and described as a gateway or point of connection, initiating conversations to identify client needs and connect them with additional support services.

**Table 2:** Summary of two major themes identified from thematic analysis emerging from interviews

Major themes	Exemplary quote/s
Dignity and respect guide all services	‘…. it’s yeah, offering people the same respect and dignity that you want to anyone else on the street, if not more, … because they’re not in a place of privilege………. I want people to feel valued and empowered and have dignity’. (ID05 – Not for profit, local community organisation) ‘just because you are experiencing homelessness or don’t have a lot of money doesn’t mean you have to eat, you know, tasteless stuff, dignity and value, really high values of ours, really high priorities’. (ID06 –Faith-based, national organisation) ‘our bottom line is if we wouldn’t eat it, we don’t expect anyone else to eat it.…. All of our food is donated, all the ingredients. come back every day are sorted and graded … and so the very best of that food goes into our community support space where people can actually go in and we don’t tell them what they should have, they can choose what they want to eat. So they have that dignity of choice…… And they start to see that they’re being valued by the food that you’re presenting to them. It’s quite common for people to walk into our space in burst into tears…. Our community gets the best that we can possibly give to them’. (ID05 – Not for profit, local community organisation)
Food serves as a gateway	‘Like you see them it’s good. It’s a good avenue to kind of like an icebreaker as well and kind of can lead into other conversations’. (ID01 – Faith-based, national organisation) ‘And now we’re going to put in telehealth, we’re going to start to bring in other allied health services, so a bit of a wraparound service for them. Because what we’re creating is a trusted space. We don’t have to be experts. We feed, have the conversations, gain their trust, and then they do allow others to come and speak to them’. (ID02 – Faith-based, local community organisation)

**Table 3:** Summary of five major subthemes identified from thematic analysis emerging from interviews

Sub-themes	Exemplary quote/s
A platform for social connection and participation	‘they have a proper dining room… feeding them and allowing them to communicate with each other and have some fun and actually enjoy each other’s company…. they love that stuff. Get them off the street, let them have a bit of peace a safe place to be. They can use the bathroom facilities, they can talk to people’. (ID03 – Faith-based, local community organisation) ‘…. it’s the social interactions that come over having meals together. For a lot of people, it’s actually really lovely to see they sit and you can see that they know that their safe, and this is their place to be and their eating, eating well… But it also then allows for other conversations to happen around what else they need’. (ID07 – Faith-based, local community organisation) ‘volunteer led mentoring service. So that’s, you know, people come in and we train them up. And then we match them with people in our community who are just needing a friend, but also someone who can help them, make some goals and then find reach those and so they connect’. (ID04 – Faith-based, local community organisation)
Servicing clients with diverse needs and experiences	‘a big part of what we do is telling peoples’ story, people who can’t tell their story, making sure that it gets out there…. telling the stories, trying to, I guess, shift the concept that there’s a particular type associated with those needing to access food relief around substance abuse or around unemployment, that it’s actually it’s not people living on the street, it’s people living in your street’. (ID12 – Not for profit, national organisation) ‘I don’t need to know your story, I just, you’re hungry, you’ve come and asked for food. Let me help you with that…. I’m like, I just want to be very careful that if I offer you a budgeting course, because you’ve come to us for food, I’m actually telling you, I’m assuming you can’t budget but I don’t think that that’s necessarily always the case. I’m also going to suggest that it just because you can’t cook something doesn’t mean you can’t cook something. And there may be a million reasons why you can’t cook something and you may not want to tell me’. (ID06 – Faith-based, national organisation)
Dependence on staff and volunteers with varying knowledge and skillsets	‘we have two staff about 30 volunteers’. (ID02 – Faith-based, local community organisation) ‘we actually had a volunteer Chef come in, and teach and just do workshops and that was fantastic. So we’ll just, we’ll just take him up anything that we provide…. So like, it’d be great to have them here all the time, but we understand when they can’t come. Because it’s like they’re volunteering their time’. (ID01 – Faith-based, national organisation) ‘….…. most volunteers actually have real jobs and have limited capacity and time to, to, to do to be responsible for some of these things, or a limited capacity to do it for a long period of time. So once they move on, you’ve got to train someone else, and so on and so forth. And you lose that knowledge’. (ID03 – Faith-based, local community organisation)
Spectrum of services, resources and facilities	‘we’re not funded and when everything in {location] was closed during COVID, everything was closed. We weren’t. …. And the only reason we could do that was because we’re not funded, or the government departments were shut down’. (ID02 – Faith-based, local community organisation) ‘I also think that government throwing money at particular funding of purchasing of food brings its own inherent, it brings its own challenges and risks as well. …. if there is too much control, you lose your ability to bid on it dynamic and responsive in a field like this, which you have to be able to be dynamic and responsive’. (ID12 – Not for profit, national organisation) ‘I would like to offer every one of our guests that come in a choice of meals, you know, would you like chicken or fish, would you like, you know, if you sit at the front of the plane, you get the chicken, if you sit at the back, you usually get the fish. So if you’re the last thing, you’re likely to be eating fish, but it is what it is… from a budgetary perspective, it’s very, very difficult to, to be able to do that’. (ID06 – Faith-based, national organisation)
Necessity of community collaboration	‘better collaboration, less organisations doing the same thing, if we could just get everyone’s swimming together and cut out all the little guys and actually focus on doing bigger and better, I reckon we’d get a better outcome’. (ID03 – Faith-based, local community organisation) ‘we could do better is as a community is to have a shared, resources are an issue like I’m talking real estate I’m talking refrigeration storage, to have more in like we’re regional area, and not a city area’. (ID05 – Not for profit, local community organisation) ‘because of all the pressing needs, I would love to, for us to be able to support that as well. I would love to see inter referrals between agencies, especially, I guess, in areas where we can’t quite where we don’t have that kind of specialisation. So drug and alcohol use domestic violence, and homelessness, I would love to see more into agency relationships and referrals happening’. (ID11 – Faith-based, local community organisation)

**Fig. 1:**
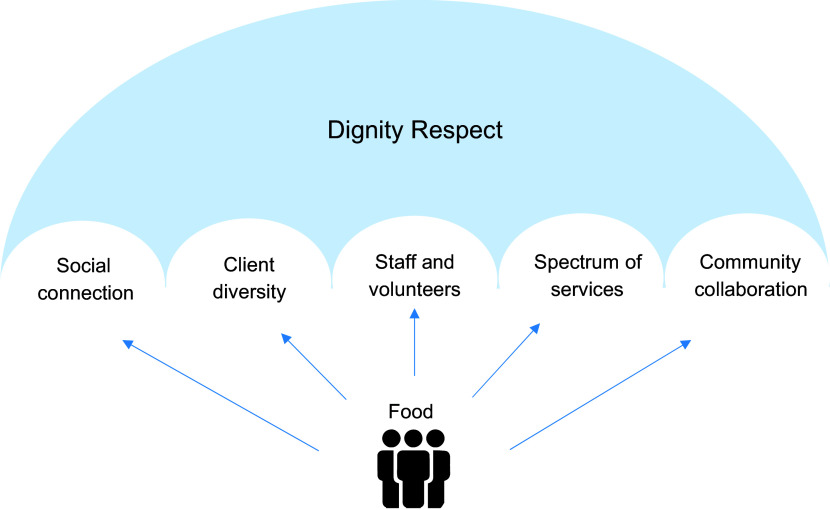
Themes emerging from interviews: food-related support for people experiencing disadvantage

The five remaining subthemes highlighted the social value of ‘food as a platform to reduce social isolation, foster connection and promote participation’, challenges in ‘servicing clients with diverse experiences and needs’, ‘dependence on staff and volunteers with varying knowledge and skillsets’ to ensure ‘adequate access to services, resources and facilities’ and the ‘necessity of community collaboration’ to provide holistic support (Fig.1).

## Dignity and respect guide all services

All participants emphasised the importance of dignity and respect for clients in their provision of food support. Participants expressed that these values play a vital role in mitigating the negative effects of socially constructed stigmas and stereotypes that individuals seeking food relief often face, which commonly create feelings of shame, embarrassment and judgement. This philosophy was applied to ensure that services and environments were non-discriminatory, inclusive and accessible to all. Dignity and respect also ensured that staff and volunteer interactions came from a place of understanding and empathy, fostering a sense of trust and safety, allowing individuals to engage with services without experiencing fear of judgement.

Participants further emphasised the importance of offering services that prioritise autonomy and choice for clients. This involved empowering individuals to make decisions, ensuring that their preferences, dietary needs and cultural considerations were respected and accommodated to the greatest extent possible. By promoting autonomy and choice, service providers aimed to enhance the overall experience of clients and avoid any sense of disempowerment or dependency. For example, food, meal services and environments were often reported to be set up to make the clients feel like guests and to provide as much choice as possible. This directly influenced food environments with multiple services having a café style setup where clients could sit down and be waited on. Some food pantries were set up like supermarkets where clients were able to select their items to allow for culturally safe/appropriate foods, food intolerances, allergies and individual taste preferences. This design feature was also reported to reduce food waste as clients could choose what they would consume.

Dignity and respect also directly impacted the quality and variety of foods accepted and therefore provided by services, despite being largely reliant on food donations. For example, interviewees described the importance of creating meals that are of high nutritional quality, wholesome, comforting and ‘filled with love’ to ensure their clients felt valued.

## Food serves as a gateway

The second overarching theme was in relation to food transcending a fundamental human right that is integral to life, serving as a gateway for conversations and connection as well as an enabler to provide additional support pathways. Food and meal provision were described to create distinctive and indirect opportunities for individuals to establish connections with both organisational staff and other service users. All participants acknowledged that, although many individuals sought these services primarily for food/sustenance, they also served as a means to alleviate social isolation. Ongoing conversations initiated during these interactions facilitated the development of trust, rapport and relationships at a pace that was comfortable for clients.

Specifically, the provision of food environments offered a platform for exploring specific needs, barriers and goals of clients. This presented organisations with opportunities to connect their clients with additional services and support within their organisation or via referral pathways. Where possible, food and/or meal services were provided in collaboration with other comprehensive support services, such as access to laundry and bathroom facilities, provision of hygiene packs, availability of health services (including community nursing, dental care, health screenings, counselling and mental health support), with the advantage of being offered within a secure and familiar environment. Support programs included budgeting and financial skills, mentorship services, guidance on matters related to family violence, psychosocial support and educational and employment opportunities, including job readiness training.

## A platform for social connection and participation

The first subtheme identified was the social value of food. Participants described that many of their clients were lonely and socially isolated and that their services went beyond providing meal. Similar to connecting clients with additional support services, food environments were described as creating a safe space to reduce isolation through opportunities for social and emotional connection. As well as communal or shared dining experiences and/or community gardens were identified as important places where clients could have fun, converse, connect, build relationships and even develop friendships with their peers.

Moreover, this provided an opportunity for clients to support each other through sharing knowledge and experiences with their peers. Some services intentionally offered group therapy and mentoring services as a way of reducing social isolation, fostering connection and building friendships.

Some organisations integrated client participation directly into their provision of food services, such as opportunities for clients to volunteer in the food pantry or engage in post-meal service clean-up. This active involvement often resulted in clients experiencing a sense of inclusion, purpose, community and empowerment, leading to their transition to becoming volunteers themselves and gaining skills for future employment.

## Servicing clients with diverse needs and experiences

The second subtheme describes the diverse experiences and needs of clients. Participants highlighted a large diversity in the dietary, health and social needs of individuals seeking assistance, which presented challenges for service provision. For example, although the importance of social connection was identified, it was acknowledged that some clients felt uncomfortable in larger centres, within large groups of people. Needs range from those living with health conditions, such as malnutrition, cancer, poor dentition, allergies and diabetes, to those with severe mental health disorders, substance and/or alcohol abuse, to people experiencing homelessness and/or family and domestic violence issues and those individuals experiencing an unexpected crisis or financial distress.

Notably, organisations reported an increasing number of individuals/families accessing services for the first time as a result of financial distress, particularly since the COVID-19 pandemic, rises in inflation, and subsequent increases in the cost-of-living expenses. Many participants reported that their organisations had provided additional weekend services to cater for increasing numbers of working individuals and families who were typically unable to attend weekday services due to work/study commitments.

Organisational staff emphasised that despite client diversity, support was always guided by dignity, respect autonomy and choice to encourage individuals to feel valued and foster their trust through rapport building. Individual choice was also respected regarding a person’s level of engagement with available services, without imposing any preconceived notions or biases.

## Dependence on staff and volunteers with varying knowledge and skillsets

The next subtheme identified was the reliance on a very limited number of employed staff and transient volunteers with diverse knowledge and skillsets. This subtheme closely relates to the issue of ensuring adequate access to services, resources and facilities. Insufficient financial resources were a constraint that hindered an organisation’s ability to employ staff with specialised skillsets, limiting their capacity to optimise operations. A heavy reliance on transient volunteers presented additional challenges for organisations in providing and enhancing services.

Participants also highlighted a heavy dependence on the existing knowledge and skills of staff and volunteers. They acknowledged the benefits of having incidental professionals such as chefs, executive management and health professionals within their volunteer teams. However, limitations in effectively utilising volunteer expertise and sustaining professional-led services were noted. In particular, a lack of long-term commitment, financial and time constraints posed obstacles to maintaining programmes and services, hiring permanent staff and hindered opportunities for upskilling, training and development, thereby limiting the quality improvement and sustainability of services provided.

Despite the challenges in fully harnessing the expertise of volunteers, participants expressed overwhelming gratitude towards them. Some participants acknowledged that a lack of knowledge and skills among staff/volunteers posed challenges to productivity and efficiency. Examples included a lack of ability to utilise available ingredients in meal planning at short notice and the provision of misinformation to clients, despite good intentions.

Participants provided ideas related to key training areas that they felt would be most effective for their staff and volunteers to improve current services. The most common suggestions included training related to nutrition frameworks/guidelines, strategies to prevent food waste, working with a broad range of ingredients, food safety issues and catering to special dietary requirements.

Notably, while all participants acknowledged not receiving specific nutrition training, except for food safety training, their primary focus remained on providing nourishing and ‘healthy’ foods, meals and hampers.

## Spectrum of services, resources and facilities

The next subtheme described the broad spectrum of ‘services, resources and facilities among different organisations’. While some organisations reported having ample resources and well-equipped facilities, others faced obstacles due to limited equipment, storage space and budgets. The availability of services, resources and facilities was found to directly impact both client access and the scope of services provided. For example, some organisations (usually affiliated with larger church groups receiving some financial support) reported having large-scale commercial kitchens producing thousands of meals per week and offered wrap-around support, while other services had minimal equipment and storage facilities, with one participant reporting running a meal service out of a food trailer in the park and using volunteers’ residential homes for storage of food and equipment. To address issues of poor facilities, some organisations proposed the establishment of shared government-funded storage spaces.

Participants were typically dependent on charitable financial and food donations to run their services, however, emphasised the importance for their organisation to maintain their independence. To continue to be adaptive and dynamic based on community and client needs, most described actively avoiding seeking government funding and support, due to the control, ‘red tape’ and rigidity it brings.

Participants further noted that insufficient funding and a high reliance on food donations pose challenges for volunteers in planning and preparing nourishing meals, given the unpredictable and highly varied produce and quality of donated food. Participants reported the need to be adaptive and creative in using a broad range of ingredients on short notice. As a result, food provision services prioritise meals that are quick and easy to prepare, affordable/budget-friendly and can be utilised, stored or frozen appropriately. This is particularly important for clients who may have long commutes or lack access to cooking and storage facilities.

## The necessity of community collaboration

The final theme depicts the necessity of community connection and collaboration among organisations to effectively support the broader needs of clients. This is key for already stretched services to ensure resources are offered and utilised as efficiently as possible. Improving communication within local communities and between agencies to develop a shared understanding of existing services (including availability) was commonly suggested as something that would be helpful for both organisations in providing adequate, differentiated and necessary services towards supporting their clients. Examples suggested included ensuring meal services were offered at different times on different days or that clients had access to culturally appropriate food on days when a particular service is not open by linking them with alternative options. Particularly for smaller communities, it was suggested that collaboration could be enhanced through joint operations including food and meal services sharing facilities such as food collection and distribution, refrigeration and storage spaces.

This integrated approach was described as particularly important for clients facing challenges or seeking additional support beyond the scope of a specific service. The importance of being able to offer and provide continued access to holistic support through current services, inter-agency support or referral pathways, extending beyond crisis intervention, was also emphasised, particularly for clients with highly complex needs.

As previously mentioned, food services play an important role in fostering conversations and creating opportunities for staff and volunteers to link their clients with other support services. However, to do so effectively, they require knowledge of existing services and community connections. One participant highlighted the benefit of being involved in a community coalition, which included individuals from the local community, as well as government and non-government organisations. With support from the local council, this coalition has developed a comprehensive handbook outlining a wide variety of local organisations available within the area including Indigenous support, accommodation, advocacy, animal welfare, child services, health, crisis support, counselling, migrant support, employment, housing, legal assistance and more. The handbook was described to be an invaluable resource due to its significance and usefulness in guiding referrals and providing essential information and frequently offered to and referenced by volunteers during conversations with clients who attend their local meal service. A directory of services was identified by other organisations that did not have access to a similar handbook as an opportunity to better support their clients. For some, collaboration was reported to be challenging at times, sometimes even bringing a sense of competition, not comradery between organisations.

## Discussion

This study explored factors that may influence the provision of food support to people experiencing disadvantage, from the perspectives of individuals representing a range of service providers/organisations. The importance of dignity, respect (including respect for the social value of food) and inclusion were identified as key elements of effective service provision^(30,31)^. Service providers were perceived to be in a unique position to offer effective support and assistance to vulnerable people with highly diverse needs and experiences, mainly because they are their primary point of contact. As such, service providers have the opportunity to identify and address clients’ needs through direct provision of comprehensive support and resources, or through referral to other services as required by individual clients. However, to effectively facilitate holistic support, study participants agreed that service providers require additional staffing, training, resources, facilities, community relationships, collaboration and coordination with other services.

The Universal Declaration of Human Rights and the WHO Constitution (1946) affirms that all individuals possess equal dignity and rights, including the right to food and healthcare without discrimination^(32,33)^. Despite this, individuals accessing charitable food and community health services frequently experience ‘undignified care’, which consequently erodes their dignity^(30)^, negatively impacts their physical and mental health^(34)^ and fosters feelings of embarrassment and shame potentially leading to a reluctance to seek help^(35)^. Promoting dignity and respect in these services requires inclusivity, non-discrimination and accessibility for all^(26)^, alongside minimisation of barriers to engagement, facilitation of social interaction, regular engagement in dignified communications between service providers and users and provision of high-quality food which is palatable, visually appealing and nutritious.

A number of barriers to service provision were identified, including a lack of trained personnel or over-dependence on volunteers with often inadequate knowledge and skills. Minimal opportunities for training were also identified as barriers, especially in dealing with high numbers of people affected by trauma-related experiences^(36)^. Implementation of trauma-informed care and resilience-informed approaches to promote the health and well-being of people experiencing disadvantage is therefore imperative^(37,38)^. Providing training for staff and volunteers in mental health first aid and/or trauma-informed care (including personal empowerment and financial education) could further promote dignity and respect. This may also afford additional benefits such as reduction in depressive symptoms, household food insecurity and poverty, especially for families participating in public assistance programs^(39)^. To address barriers associated with limitations in training opportunities, previous studies have demonstrated success with frameworks that build upon the capability and capacity of the existing charitable food services workforce by involving the university sector to support training in health and nutrition education (such as students-in-training on placements)^(40)^. These initiatives have proven to be both inexpensive and mutually beneficial.

Priortising client autonomy and choice was also identified as a strategy to promote a sense of control, social inclusion, empowerment and independence in people accessing some services. This includes allowing people to make choices, such as selecting their food items or offering a variety of options at community meals. Social supermarkets, which are food-oriented retailers selling products to a restricted group of people living in or at-risk of poverty, have gained significant popularity worldwide^(41)^. These establishments have been shown to successfully support socially disadvantaged groups by providing them with choices in food options, and hence preserving their dignity. A recent evaluation of the potential for implementing a social supermarket model in Australia, combining elements of community food relief services, social enterprise, social services and employment pathways, has yielded promising results.^(42)^ The implementation of this model was relatively straightforward and had positive outcomes, such as fostering social connections and increasing accessibility to nutritious food options, consistent with the goals of providers in our study.

Study participants highlighted urban agriculture, particularly community farms and gardens, as a promising approach to address food inequities in people experiencing disadvantage and to yield other benefits across social, economic, environmental, cultural and health domains^(43)^. These initiatives not only enhance social connection but also improve access to fresh produce and thereby, food quality, while also addressing systemic challenges and driving essential structural changes for broader nutritional and health benefits. This is particularly important considering the escalating prevalence of severe food insecurity in Australian households, reaching 21 % in 2022, up from 17 % in 2021^(44)^. This increase in food insecurity is linked to rising living expenses (e.g. food and energy prices), reduced or low income and limited government benefits^(44)^, all of which disproportionately impact the most vulnerable in society^(45)^. Notwithstanding, food insecurity is associated with negative health outcomes, including higher prevalence of hospitalisation, depression and chronic diseases and exacerbates diet-related health inequalities^(39)^.

To enhance diet quality in charitable food systems, study participants called for the development of nutrition-specific policies, standards or guidelines. Food safety laws in Australia are regulated by the Australia New Zealand Food Standards Code (FSANZ). While the code does not explicitly address donated food, it permits the adjustment of labeling to rectify mislabelling issues and allows for the sale or donation of food after the best before date, provided appropriate adjustments are made to ensure food safety^(46)^. Beyond this, the Australian food relief sector has no unanimous regulation or compliance. Hence, newly developed nutrition standards and accompanying training tools for staff and volunteers could potentially facilitate the attainment of adequate nutritional quality for clients of charitable organisations in a cost-effective way. The nutrition standards could be based on those already in use in healthcare facilities^(47)^ or home-delivered and centre-based meal programmes^(48)^, which are holistic in nature and encompass accommodation standards and educational toolkits^(49,50)^. The nutrition standards would ideally extend beyond nutritional adequacy to consider client psychosocial needs and would involve service users in the menu design (including menu cycles) and selection of food items^(47)^ (including minimum numbers of food choices).

It is established that charitable organisations which offer food services are in a unique position to expand beyond food provision to include preventative health and social services^(51)^. Evaluations of community-based health and social services, or programmes blending social and primary care, have demonstrated positive health outcomes^(52)^. These programmes often utilise wraparound support delivery models and multi-service, holistic approaches and may be of particular benefit to people who may be wary of engaging with formal systems of care. Integrating preventative health services into charitable organisations also represents a cost-effective solution for reducing Australia’s economic disease burden, improving access to health services, and long-term improvements in dietary-related public health outcomes^(23)^. This approach also aligns with the Australian Government’s *National Preventative Health Strategy 2021–2030* ^(22)^. Integrated food relief systems that partner with hospitals and community outreach programs could also provide holistic solutions.

Enhanced community collaboration was identified as an essential opportunity for sharing services, establishing referral pathways and maximising service effectiveness. While challenging to implement, collaborative practice^(53)^ and inter-agency collaboration^(54)^ have increasingly been recognised as important strategies to coordinate healthcare services and achieve positive outcomes. Such collaboration has the potential to improve service delivery for individuals requiring multiple services and allow for more efficient utilisation of often insufficient services, resources and facilities. The provision of support has the follow on effect to streamline nutrition services to those who may need them. To foster community collaboration and improve service accessibility, some study participants suggested the creation of a centralised directory listing diverse local services/organisations. This directory could include services such as Indigenous support; accommodation; advocacy; animal welfare; child services; health; crisis support; counseling; migrant support; employment; housing; legal assistance and more. This resource would be especially valuable for individuals with specific nutritional needs, as it facilitates tailored support from relevant organisations.

Despite participants identifying some solutions to improving current support services, evidence demonstrates that charitable food systems, both globally and in Australia, do not effectively address the underlying causes of food insecurity and cannot solely bring about systemic change^(55,56)^. While charitable systems are well intentioned, they fail to tackle the root causes and the broader drivers of the issue^(57)^. This highlights a need for comprehensive and systemic changes to effectively address diet-related health inequities attributed to food poverty, extending beyond the scope of charitable efforts alone. It is important to avoid inadvertently reinforcing the perception that addressing food poverty is solely an individual or community responsibility, as this deflects from the need for government intervention and political action^(56,57)^.

Improving access to food and health services, however, serves as an important first step in addressing food insecurity and diet-related health inequalities. Commissioned by the South Australian Government, as part of the South Australian Food Relief Charter^(58,59)^, has taken a significant step towards supporting food relief services to going beyond food provision to address broader drivers of food insecurity and improving equitable access to nutritious foods. This has involved creating nutrition guidelines and a commissioned policy–research–practice collaboration to develop support pathways to address the root causes of food insecurity. Such collaborative approaches have the potential to be extended or replicated in other geographical areas to enhance community connections and support client needs more efficiently.

One notable project aligning with the charter’s principles is the Social Supermarket expansion project^(42)^. This initiative explores the potential of local blended service models in South Australia, combining food, employment pathways, social services and social enterprise components in single-site hubs or collaborative inter-agency alliances. The Social Supermarket model, characterised by well-defined criteria and language, provides a framework for organisations transitioning from emergency food relief, as evidenced by positive outcomes in pilot studies. However, it is important to highlight that there has been insufficient time to conduct a comprehensive evaluation of the outcomes associated with the charitable food sector.

## Limitations

Although this study explored barriers and suggested solutions to improve services for supporting the nutrition-related health and well-being of people experiencing disadvantage, these findings are limited to the perspectives of interviewed service providers, who may not fully represent the diversity within the entire sector engaged in providing food and health support. This study is part of a larger study including varied stakeholders, but this work alone lacks the perspective of people accessing services, therefore these results are not generalisable to the broader sector.

Acknowledging the significance and advantages of tailoring interventions, food and healthcare services to the specific needs of communities, it is recognised that a one-size-fits-all approach is unlikely to be effective in this population, given the diverse needs and lived experiences of individuals accessing these services. Given the small sample size and the motivated nature of participants, findings may be subject to participant, social desirability and content bias, which should be taken into account when interpreting results. However, the interview guide was based on a previous review of the literature and survey results to design relevant questions. Future research will explore the perspectives of people accessing services aiming to identify insights for development and redesign of services, identifying barriers and solutions for consideration in future service interventions.

## Conclusion

People experiencing disadvantage face significant challenges in accessing nutritionally adequate diets and appropriate health services, leading to disparities in diet-related health outcomes. Dignity and respect are core values that service providers strive to maintain in their service provision, and they recognise the importance of creating opportunities for social interaction for their clients. Food and sharing meals is part of that interaction. However, service providers acknowledge they cannot fully address the underlying factors contributing to their clients’ health inequities, including limited access to nutritious food and healthcare. Service providers involved in food-related support are in a unique position to identify individual needs and implement holistic preventative health interventions. To achieve this, a trained and sustainable workforce, shared resources and facilities are required. To effectively connect individuals with the necessary supports, it is essential to establish strong community relationships, foster collaborations and enhance coordination between service providers. Future interventions should focus on finding solutions to improving accessibility to high-quality food and health services while prioritising the integration of dignity, respect and social connection as essential components of their design.
